# Tetraspanins, Another Piece in the HIV-1 Replication Puzzle

**DOI:** 10.3389/fimmu.2018.01811

**Published:** 2018-08-03

**Authors:** Henar Suárez, Vera Rocha-Perugini, Susana Álvarez, María Yáñez-Mó

**Affiliations:** ^1^Departamento de Biología Molecular, Universidad Autónoma de Madrid, Madrid, Spain; ^2^Servicio de Inmunología, Hospital de la Princesa, Instituto de Investigación Sanitaria La Princesa (IIS-IP), Madrid, Spain; ^3^Vascular Pathophysiology Research Area, Centro Nacional de Investigaciones Cardiovasculares Carlos III, Madrid, Spain; ^4^Servicio de Inmunobiología Molecular, Hospital General Universitario Gregorio Marañón, Madrid, Spain; ^5^Centro de Biología Molecular Severo Ochoa, Instituto de Investigación Sanitaria La Princesa (IIS-IP), Madrid, Spain

**Keywords:** tetraspanins, HIV, entry, assembly, budding, reverse transcription

## Abstract

Despite the great research effort placed during the last decades in HIV-1 study, still some aspects of its replication cycle remain unknown. All this powerful research has succeeded in developing different drugs for AIDS treatment, but none of them can completely remove the virus from infected patients, who require life-long medication. The classical approach was focused on the study of virus particles as the main target, but increasing evidence highlights the importance of host cell proteins in HIV-1 cycle. In this context, tetraspanins have emerged as critical players in different steps of the viral infection cycle. Through their association with other molecules, including membrane receptors, cytoskeletal proteins, and signaling molecules, tetraspanins organize specialized membrane microdomains called tetraspanin-enriched microdomains (TEMs). Within these microdomains, several tetraspanins have been described to regulate HIV-1 entry, assembly, and transfer between cells. Interestingly, the importance of tetraspanins CD81 and CD63 in the early steps of viral replication has been recently pointed out. Indeed, CD81 can control the turnover of the HIV-1 restriction factor SAMHD1. This deoxynucleoside triphosphate triphosphohydrolase counteracts HIV-1 reverse transcription (RT) in resting cells *via* its dual function as dNTPase, catalyzing deoxynucleotide triphosphates into deoxynucleosides and inorganic triphosphate, and as exonuclease able to degrade single-stranded RNAs. SAMHD1 has also been related with the detection of viral nucleic acids, regulating the innate immune response and would promote viral latency. New evidences demonstrating the ability of CD81 to control SAMHD1 expression, and as a consequence, HIV-1 RT activity, highlight the importance of TEMs for viral replication. Here, we will briefly review how tetraspanins modulate HIV-1 infection, focusing on the latest findings that link TEMs to viral replication.

## The Cellular Plasma Membrane as the First Modulator of HIV-1 Infection

HIV-1 virus belongs to Lentivirus within the RNA family *Retroviridae*. It carries two identical molecules of positive ssRNA that are converted to dsRNA intermediate by viral RNA-dependent DNA polymerase (reverse transcriptase). HIV genome encodes for 16 proteins participating in several phases during the HIV life cycle, the structural polyproteins Gag [consisting of matrix, capsid, nucleocapsid (NC), and p6 proteins], Pol (consisting of protease, reverse transcriptase, and integrase), and envelope (Env; gp120 and gp41); regulatory proteins (Tat and Rev); and accessory proteins (Vif, Vpr, Vpu/Vpx, and Nef) (1).

The HIV-1 envelope glycoprotein (Env) facilitates viral attachment and entry into host cells (2). Three spikes form the Env trimeric complex, each spike consisting of the association of a gp120 subunit on the surface and a transmembrane gp41 molecule (3). Gp120 interacts with CD4, the cellular transmembrane receptor expressed on the membrane of the target cell; and this induces a conformational change in gp120 that exposes new sites for co-receptor binding. There are two types of HIV-1 viruses regarding co-receptor preference, either CCR5 and/or CXCR4. After this second interaction, a hydrophobic region in gp41 is exposed and inserted into the plasma membrane, so that viral and cellular membranes get close enough to create the fusion pore (2, 4). Besides the receptor and co-receptor, other cell surface molecules expressed on dendritic cells (DC) can act as attachment factors, although they do not trigger viral fusion. Most attachment factors are C-type lectins, or calcium-dependent glycan-binding proteins such as DC-SIGN, Siglec-1, mannose, langerin, or DCIR (5–7).

The plasma membrane is not a homogeneous surface but contains specialized microdomains that can be differentiated by their composition and function: lipid rafts, tetraspanin-enriched microdomains (TEMs), caveolae, and clathrin-coated pits (8, 9). Lipid rafts, enriched in cholesterol and saturated lipids with long hydrocarbon chains and hydroxylated ceramide backbones (10–12), provide an environment that favors the inclusion of oligomeric proteins such as flotillins and caveolins, or proteins with lipid modifications such as palmitoylation or GPI anchors (13–15). While lipid rafts properties rely mainly on their lipid content, TEMs are organized around protein–protein interactions nucleated by tetraspanins (9). Tetraspanins, a superfamily of ubiquitous four transmembrane proteins, laterally interact with other membrane molecules stablishing specialized domains or platforms called TEMs. The most common partners of tetraspanins are integrins, proteins of the immunoglobulin superfamily, metalloproteinases, membrane receptors, and other tetraspanins (9). TEMs also include cholesterol and gangliosides. Lipid–protein and protein–protein interactions are facilitated by multiple palmitoylation sites in both tetraspanins and their partners (16).

**Figure 1:**
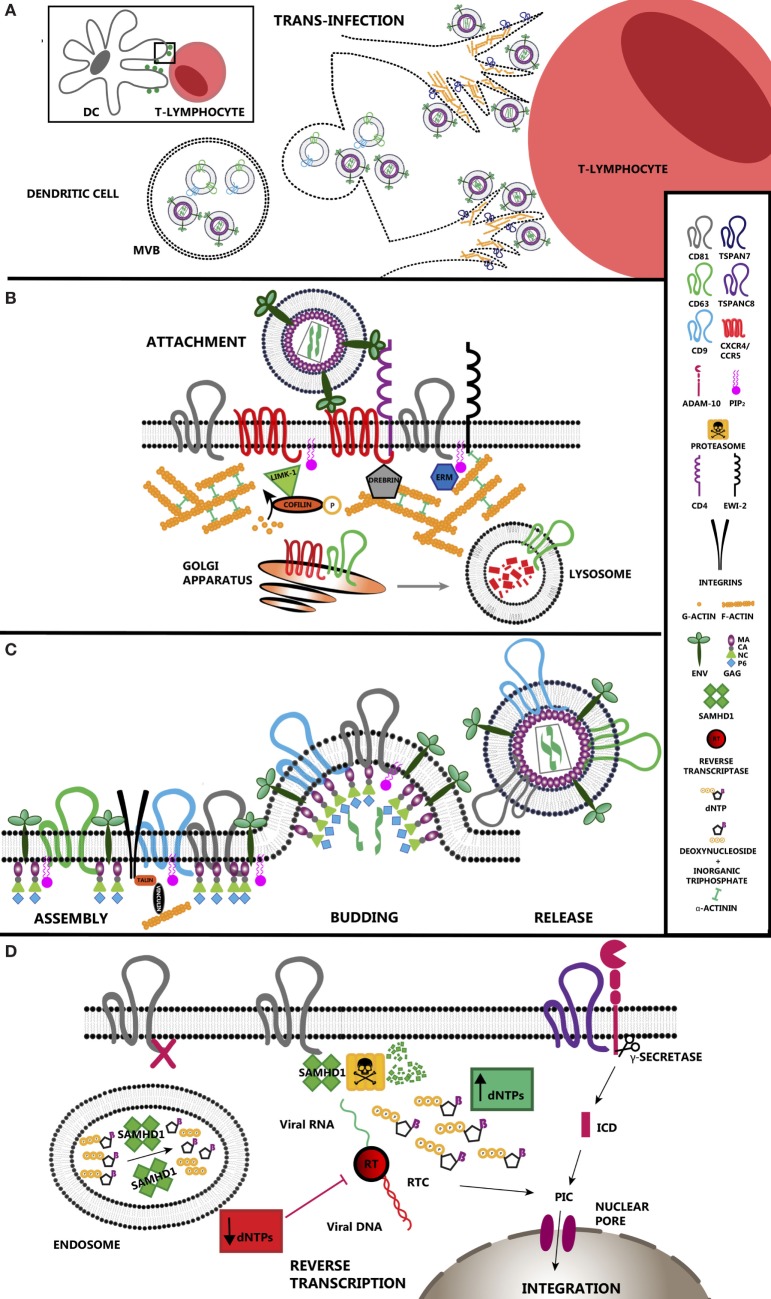
Tetraspanin roles during HIV-1 infection. **(A)** Tetraspanins regulate transinfection of T-lymphocytes. Dendritic cells stablish contacts with T-lymphocytes during antigen presentation. HIV-1 takes advantage of immune synapses to enhance the infection of T-lymphocytes, the main target cells for the virus. This strategy is called trans-enhancement or transinfection and takes place through two different pathways. One involves the endocytosis of viral particles by DCs, which gives them access to endosomal compartments. As happens with exosomes, viral particles accumulate in multivesicular bodies that finally fuse with the plasma membrane releasing those particles together with exosomes into the intercellular space. The second pathway involves TSPAN7, which inhibits viral endocytosis and promotes formation of actin rich protrusions in DCs. In this scenario, viral particles are sequestered on the surface of these cells, allowing virus exposure and transfer to T-lymphocytes. **(B)** TEM regulation of HIV-1 entry. CD4 and co-receptors CCR5/CXCR4 segregate within tetraspanin-enriched microdomains (TEMs), which control their proper distribution and dynamics enhancing HIV-1 attachment efficiency and subsequent entry. CD63 regulates the expression of CXCR4 on the cell surface by stimulating its degradation through the lysosomal pathway. Env binding to its receptor and co-receptor brings them closer and triggers several intracellular pathways where actin polymerization is the main response. Active LIMK1 phosphorylates and inactivates cofilin, stimulating actin polymerization. Proteins such as moesin or α-actinin have a structural function as they link receptors and tetraspanins to the subcortical actin network. Other proteins such as drebrin control the stability of the actin web. TSPAN7 is also a positive regulator of actin polymerization, although the effectors downstream have not been addressed yet. **(C)** HIV-1 assembly occurs at TEMs. Viral protein Gag interacts with the inner leaflet of the plasma membrane *via* its myristoylation, which increases the affinity for cholesterol-enriched areas. Gag also interacts with the positively charged PIP2 and the inner loop of different tetraspanins such as CD81 and CD82. Gag induces CD9 clusterization. However, there is no direct evidence indicating an essential requirement for tetraspanins during HIV-1 budding. Recruitment of all these components into restricted areas may involve the presence of the subcortical actin web for their stabilization, where talin and vinculin would act as a link. **(D)** HIV-1 reverse transcription (RT) is regulated by tetraspanins. SAMHD1 is a negative regulator of viral RT as it decreases the concentration of deoxynucleotide triphosphates available in the cell. CD81 regulates SAMHD1 activity by stimulating its degradation *via* proteasome. CD81 depletion induces the relocalization of SAMHD1 inside early endosomes. ADAM-10 activity is regulated by tetraspanin TSPANC8 subfamily. The resulting intracellular domain when cleaved by a γ-secretase has been identified recently as a component of the PIC. When RT is completed, viral DNA is transported into the nucleus where it integrates in the cell genome.

Given the complex structure of the plasma membrane, it is not surprising that the CD4 receptor and CCR5/CXCR4 co-receptors are not randomly distributed on the cell surface, but show a controlled segregation pattern into defined membrane clusters (17). This enrichment in specialized microdomains has been also reported for attachment factors such as DC-SIGN, in the surface of DCs (18). Inclusion of HIV-1 receptors and co-receptors in lipid rafts, caveolae microdomains, or TEMs tightly regulate viral entry. Since the presence of cholesterol is a common feature of these different microdomains, its depletion or the use of antibodies that specifically recognize clustered cholesterol on the cell surface induces a reorganization of the plasma membrane, disrupts receptor clustering and membrane dynamics, and inhibits virus entry (19, 20). These antibodies do not appear to mask CD4 and CXCR4 interaction sites, but rather seem to affect CXCR4 membrane diffusion, triggering an excess of CD4-CXCR4 clustering, which prevents proper attachment of the viral envelope proteins (19). CD4 and CCR5 co-receptor interact with each other under basal conditions, and addition of gp120 protein bring them closer (17, 21). Tetraspanins CD81 and CD82 also associate with the CD4 receptor on T-cells (22, 23), and gp120 attachment to CD4 induce co-clustering of CD81 (24). CD81 modulates CD4 dimerization and clustering, and it decreases CD4 ability to bind to gp120 (25). All these results support the notion that membrane microdomains are critical regulators of HIV-1 receptors diffusion, allowing proper clustering and efficient protein–protein interactions required for viral entry (26) (Figure 1B). Under resting conditions, lipid rafts and TEMs are mainly independent domains at the cell surface, recognized by the presence of specific markers. However, after viral infection, Gag can induce the coalescence of the two types of domains (27).

Other studies suggest that these microdomains may also be important to regulate receptor recycling and trafficking to the plasma membrane. Thus, the tetraspanin CD63 regulates CXCR4 expression on the plasma membrane of T-lymphocytes and activated B cells. Moreover, CD63 glycosylation sites are critical for the interaction with CXCR4 (28) and promote CXCR4 trafficking from the Golgi apparatus to late endosomes and lysosomes for its degradation (29, 30) (Figure 1B).

## Cytoskeleton, A Second Barrier for the Virus?

Successful HIV-1 entry and infection depends on two sequential events, proper clusterization of the CD4 receptor and co-receptors after viral attachment, and subsequent polymerization and depolimerization of the cortical F-actin meshwork beneath the plasma membrane.

Although the cortical actin web was first described as a barrier for viral entry (21) (Figure 1A), inhibition of the actin nucleation regulator ARP2/3 was shown to inhibit viral Env-induced fusion, highlighting the importance of an early actin polymerization phase that stabilizes viral attachment and subsequent fusion with the plasma membrane (31). In addition, the tetraspanin TSPAN7 has been recently identified as an effector of actin nucleation (32), necessary for the formation of actin-rich dendrites in DCs that capture, present, and transfer viruses to T-lymphocytes (33), in the process called trans-enhancement or trans-infection (Figure 1A).

Gp120 binding to CXCR4 regulates actin dynamics through the switch off and on of the actin-binding protein cofilin (21), which is inactivated by LIMK-1-dependent phosphorylation, promoting actin polymerization and receptor clustering (34). LIMK-1 is activated by CXCR4 *via* two different pathways: the Rac1/PAK and the RhoA/ROCK pathways. The activation of the latter depends on filamin-A, an actin adaptor protein that binds to CD4, CXCR4, and CCR5 (35). Although the primary activator of both pathways has not been addressed yet, tetraspanins CD82 or CD81 could be good candidates. CD82 can interact with CD4 and regulates actin dynamics in both T-lymphocytes and cancer cells through the modulation of RhoA and Rac1 signaling (36, 37), while CD81 regulates Rac activity turnover (38).

Besides Rho GTPase activity, the membrane lipid phosphatidylinositol 4,5-biphosphate (PIP_2_) facilitates viral infection by controlling the activity of several actin-binding proteins (31). Among them, ERMs (ezrin, moesin, and radixin), whose activation requires the interaction with PIP_2_ at the plasma membrane (39). Gp120 binding to CD4 receptor activates moesin, which triggers the reorganization of subcortical F-actin and stimulates CD4-CXCR4 clustering in T-lymphocytes (40) (Figure 1B). However, other studies performed in Hela cells described moesin as a negative regulator of viral infection (41), through the control of microtubule stability that could affect viral transport to the nucleus (42, 43). Moesin also interacts directly with CD81, or indirectly with either CD9 or CD81 through EWI-2, a TEM component member of the immunoglobulin family (44). EWI-2 is also linked to the actin cytoskeleton *via* α-actinin, an actin-binding protein negatively regulated by PIP2 that induces a restrictive conformation for HIV entry on the cortical actin network (45, 46). Another CXCR4 interactor, drebrin, stabilizes actin in a process dependent on the viral envelope, so that drebrin silencing increases HIV-1 entry again supporting the idea of a need for a later actin depolymerization step for viral access into the cell (47) (Figure 1B).

This later step of depolymerization is also promoted by CXCR4 through the activation of cofilin *via* Gαi signaling (48). Alteration in the levels of gelsolin, another actin regulatory molecule, also impairs HIV-1 infection (49). In this stage, destabilization of the actin network at later phases of viral entry would provide access of the virus to microtubules, which will transport the RTC [reverse transcription (RT) complex] toward the nucleus (31, 50). Studies using nocodazole (an inhibitor of microtubule polymerization), or kinesin and dynein inhibitors delay HIV-1 uncoating and promote the accumulation of viral particles far away from the nucleus. Kinesin and dynein may contribute to the uncoating process by applying opposite forces that could destabilize and disrupt the structure of the capsid while it travels through the cytoplasm (51).

Tetraspanins and the actin cytoskeleton are also crucial for DC-mediated trans-infection by which the virus is retained at or near the cell surface of a DC and transmitted to a T-lymphocyte *via* the close contact of both cells. TSPAN7 expressed in DCs is important for the formation of actin rich spikes that are able to retain viral particles on their surface (32) (Figure 1A). In addition, DCs can trap viral particles in large intracellular vesicles staining for tetraspanins CD81 and CD63 (52), although these structures may not be completely closed and remain connected with the extracellular space allowing a quick release of viral particles (53) during DC–T cell contacts. The exosome secretion pathway has been proposed as an alternative transmission route between cells without fusion events. Indeed, HIV-1 can directly use the endosomal pathway to enter DCs and be thereafter released together with exosomes after the fusion of multivesicular bodies with the plasma membrane (54, 55) (Figure 1A). In addition, recent studies suggest that in top of that, exosomes from DC are loaded with molecules that could enhance viral replication and release, such as CCR5 or CXCR4, which facilitate T-lymphocyte infection, miRNAs or viral proteins, such as Nef (56).

## HIV-1 Promotes Plasma Membrane Remodeling

Upon successful infection, HIV-1 virus can modify the cell surface of infected cells to facilitate the release of new viral particles. Vpu and Nef are the viral proteins involved in this modulation. Both of them can control CD4 expression at the cell surface by different mechanisms. Nef is synthesized during the early steps of the infection, interacting with the plasma membrane through myristoylation modifications and with the C-terminal domain of the CD4 receptor (57, 58). Nef forms a complex with AP-2, promoting CD4 endocytosis and subsequent transport to the lysosomal pathway for its degradation (59). Nef can also control MHC-I levels to protect the infected cell from the immune system, by stimulating its endocytosis from the cell surface and by inducing its accumulation at the trans-Golgi network (60). In contrast, Vpu is a transmembrane viral protein that is transcribed during the late steps of the viral cycle, blocking CD4 transport from the endoplasmic reticulum to the membrane and stimulating CD4 degradation by the endoplasmic-reticulum-associated protein degradation pathway (61).

Tetraspanins CD9, CD81, CD82, CD63, and CD231 are included in HIV-1 particles negatively regulating viral infectivity (62). How the virus regulates their inclusion into virions remains unknown, but it does not seem to be an uncontrolled process since L6, a transmembrane protein with similar topology is excluded (62). Remarkably, HIV-1 viral proteins also control tetraspanin expression on the plasma membrane. Vpu and Nef downregulate a wide variety of tetraspanins inducing their enrichment at the perinuclear region of the cell (63). T-lymphocytes from HIV-1 patients showed a reduced expression of CD82 and CD81 (64), while the expression of the latter was increased in B-lymphocytes (65). CD81 and CD82 downregulation was attributed to Vpu, and to a lesser extent to Nef. Vpu was shown to directly bind CD81, stimulating its degradation by either the proteasome or the lysosomal pathways. Although CD82 does not directly interact with Vpu, the viral protein also drives its degradation, probably through the association with CD81 (63). Therefore, downregulation of tetraspanin expression seems to be essential for virus spread. In addition, CD81 and CD9 play a negative role in viral-induced syncytia formation (24).

Viral assembly and budding is driven by Gag polyprotein, which is formed by matrix (MA), capsid (CA), NC, p6 domains, and two spacer peptides, named SP1 and SP2 (66) (Figure 1C). The initial evidence that suggested that assembly takes place in specialized microdomains came from the presence of high levels of cholesterol and sphingolipids in the HIV-1 envelope (67–69). After synthesis in the cytoplasm, Gag interacts with two molecules of viral RNA through its NC domain (70). Gag association with the cell surface then is driven by a cluster of positive amino acids in the MA domain, with affinity for negatively charged PIP_2_ in the inner leaflet of the plasma membrane. Myristoylation of the N-terminal region of the MA domain contributes to its association to membrane areas enriched in cholesterol and sphingolipids, like lipid rafts or TEMs (26, 70). Env and Gag colocalize with tetraspanins CD63, CD81, and CD9 at the plasma membrane of T-cells and direct coimmunoprecipitation of CD81 with Gag has been reported (68, 71) (Figure 1C). Moreover, in both T-cells and macrophages, there is a relocalization of CD63 from intracellular compartments to viral assembly sites; however, its depletion does not affect viral release (72) (Figure 1C). In macrophages, viral assembly takes place in vacuoles that originate from invaginations of the plasma membrane (73), and present focal-adhesion-like domains more abundant in cells infected with the virus (74). These domains are enriched in integrin β2, focal adhesion components, tetraspanins CD9, CD53, CD81 and CD82, and in PIP_2_ and AP-2, common components of clathrin-coated pits (75). After the budding of new virions, Gag is processed by the viral protease into the mature proteins enabling the formation of the capsid that contains the viral RNA genome and the enzymes needed for its replication (10, 70).

Further studies will be required to clarify the specific role of tetraspanins during the assembly and budding of new virions. All existing evidences support that tetraspanins are located at the exit sites and are incorporated in newly formed virions; however, future research should decipher whether they are functionally important for the organization and recruitment of all the components needed for budding (10).

## Intracellular Events of HIV-1 Infection are Surprisingly also Dependant on Membrane Microdomains

The binding to the viral receptor and co-receptor triggers an intracellular response that prepares the host cell for HIV-1 RT. After the fusion of the viral and the cell membranes, the capsid of the virus is released into the cytoplasm. RT occurs in a complex called RTC (RT complex), which is formed by viral proteins (reverse transcriptase, integrase, matrix protein and Vpr), the RNA genome, and host proteins needed to complete the cDNA synthesis (3, 76, 77). When the RNA genome is completely transformed into cDNA, this complex, still composed by a combination of viral and cellular proteins, is named PIC (pre-integration complex) (78). One surprising component of the HIV-1 PIC is the intracellular domain of the transmembrane A Disintegrin And Metalloprotease-10 (ADAM10) (79); so, when ADAM10 expression is inhibited, a decrease in HIV-1 RT has been reported (Figure 1D). Tetraspanins could be also involved in this event, since ADAM10 localization, trafficking, and substrate specificity is regulated by a subfamily of tetraspanins characterized for having eight Cys residues in their large extracellular loops (TspanC8) (80) (Figure 1D).

The role of the RTC in the RT process and how the uncoating process takes place is still a matter of debate. The first theory, no longer accepted, proposed that the capsid was lost immediately after membrane fusion and viral entry (81). Many studies have proven that the capsid is required for the RT process since it may provide protection against the host cell defense, as well as anchorage for the needed host factors (76). Another theory proposes that uncoating takes place while the RTC gets to the nucleus. The third one, however, claims that the whole capsid might travel along the cytoplasm until it reaches the nuclear pore complex where it is disassembled (77). There are different pieces of evidence that show that the capsid remains stable for some time after viral entry, and mutations that increase or decrease the stability of the capsid all have a negative effect on HIV-1 infection (81). Most results suggest that uncoating may occur during HIV-1 RT (82), and it should not involve a complete breakdown of the capsid but a progressive disassembly along the trip through the cytoplasm (77). Destabilization of its structure would allow the access of the nucleotides, and host proteins needed for the viral RT (83). Before nuclear entry, however, the core has to be disrupted as it is too large to cross the nuclear pore (82).

Interestingly, some data suggest that membrane-bound tetraspanins also regulate after-entry events in HIV-1 infection. CD63 has been shown to regulate HIV-1 RT, nuclear transport, and integration; however, the mechanisms involved remain unsolved (84–86). RT is also modulated by tetraspanin CD81, *via* the regulation of SAMHD1 expression (87). SAMHD1 is a deoxynucleoside triphosphate triphosphohydrolase that controls the availability of deoxynucleotide triphosphates (dNTPs) through their conversion into deoxynucleoside and inorganic triphosphate (88). Recent studies have identified an additional role of SAMHD1 in DNA repair and genome stability (89, 90). Others suggest that it may also have RNase activity over ssRNA or DNA/RNA duplexes (91), although these later results remain controversial (92). Because of its relevance, the cell has developed several mechanisms for SAMHD1 regulation. Related to its quaternary structure (93), SAMHD1 monomers associate in dimers, and these dimers organize in tetramers. The organization of SAMHD1 monomers into the active tetrameric form depends on the presence of dNTPs for its stabilization (94). Regarding posttranslational modifications, SAMHD1 can be phosphorylated by cyclin A2/CDK1 at T592 after T cell activation (95), or by tyrosine kinases downstream IL-2 and IL-7 stimulation of CD4+ T cells (96). These modifications decrease its dNTPase activity, increasing viral RT (96). Acetylation at K405 has the opposite effect, stimulating SAMHD1 dNTPase activity, and promoting the transition from G1 into S phase in cancer cells (97). SAMHD1 oxidation status is another important regulatory mechanism. Three different cysteines of the enzyme can be oxidized, changing the nucleotide binding site conformation, preventing its tetramerization and subsequent activation (98). SAMHD1 has a nuclear and cytoplasmic distribution (87, 99). Nuclear localization is mainly determined by its NLS sequence (100), and the oxidation status seems to be critical for its accumulation in the cytoplasm (98). Once in the cytoplasm, tetraspanin CD81 seems to regulate the enzyme subcellular localization into endosomes (87) (Figure 1D).

SAMHD1 expression levels are also tightly regulated. Reduced levels of SAMHD1 increase the amount of dNTPs available for viral RT. Thus, SAMHD1 is a major regulator of HIV-1 infection as it restricts the availability of dNTPs necessary for HIV-1 RT in resting monocytes, macrophages, CD4+ T cells, and DC. HIV-2 virus, but not HIV-1, expresses an accessory protein called Vpx that tags SAMHD1 for its degradation by the proteasome (93, 101). SAMHD1 interaction with the C-terminal domain of the tetraspanin CD81 also stimulates its proteasomal degradation. Depletion of CD81 abolishes SAMHD1 degradation, which is translocated into early endosomal compartments where it exerts its dNTPase activity (87) (Figure 1D). Although it is reported that HIV-1 downregulates CD81 expression at the cell surface (64), this event might only occur late in the viral cycle, after RT has been completed.

## Concluding Remarks

Tetraspanins are important regulators of HIV-1 cycle. They would have a dual role in HIV-1 infection. Tetraspanins would inhibit infectivity by actively participating in viral entry. They would modulate cell surface dynamics and the proper distribution of receptors and co-receptors, both in the host cell and in the viral membrane inhibiting viral entry and induced membrane fusion (24, 62). In contrast, CD81 can enhance viral RT by promoting SAMHD1 degradation through the proteasome, thus increasing the availability of dNTPs in the host cell (87). These opposite functions concur with a first round of active viral entry and replication to produce new viral particles in the cell, followed by a second round of viral latency to avoid recognition by the immune system so it can persist within the organism (102). HIV-1 possesses the tools to control tetraspanin expression by the host cell, avoiding undesirable effects on viral infection. As important membrane organizers, tetraspanins regulate multiple cellular proteins that control the different steps of HIV-1 infection cycle, and thus represent an interesting target for the development of new drugs against viral infection. Finally, it has been reported that peptides against the intracellular region of CD81 can block its activity over SAMHD1 and reduce viral RT (87). This result leaves open the possibility of using specific peptides against tetraspanins as an interesting strategy to restrict HIV-1 infection.

## Author Contributions

HS wrote the manuscript and designed the figures. VR-P conceived and edited the manuscript. SA commented and edited the manuscript. MY-M conceived and edited the manuscript. All authors read and approved the final manuscript.

## Conflict of Interest Statement

The authors declare that the research was conducted in the absence of any commercial or financial relationships that could be construed as a potential conflict of interest.

## Abbreviations

ADAM, a disintegrin and metalloprotease; CA, capsid; dNTPs, deoxynucleotide triphosphates; ERMs, ezrin, moesin, and radixin; ICD, intracellular domain; MA, matrix MA; NC, nucleocapsid; NLS, nuclear localization signal; NPC, nuclear pore complex; PIC, pre-integration complex; PIP2, phosphatidylinositol 4,5-biphosphate; RT, reverse transcription; RTC, reverse transcription complex; TEMs, tetraspanin-enriched microdomains.
